# Pov9D: Point Cloud-Based Open-Vocabulary 9D Object Pose Estimation

**DOI:** 10.3390/jimaging11110380

**Published:** 2025-10-28

**Authors:** Tianfu Wang, Hongguang Wang

**Affiliations:** 1 State Key Laboratory of Robotics and Intelligent Systems, Shenyang Institute of Automation, Chinese Academy of Sciences, Chuangxin Road 135, Shenyang 110016, China; 2 University of Chinese Academy of Sciences, Yuquan Road 19, Beijing 100049, China

**Keywords:** open-vocabulary, object pose estimation, point cloud

## Abstract

We propose a *point cloud-based* framework for open-vocabulary object pose estimation, called Pov9D. Existing approaches are predominantly RGB-based and often rely on texture or appearance cues, making them susceptible to pose ambiguities when objects are textureless or lack distinctive visual features. In contrast, Pov9D takes 3D point clouds as input, enabling direct access to geometric structures that are essential for accurate and robust pose estimation, especially in open-vocabulary settings. To bridge the gap between geometric observations and semantic understanding, Pov9D integrates category-level textual descriptions to guide the estimation process. To this end, we introduce a text-conditioned shape prior generator that predicts a normalized object shape from both the observed point cloud and the textual category description. This shape prior provides a consistent geometric reference, facilitating precise prediction of object translation, rotation, and size, even for unseen categories. Extensive experiments on the OO3D-9D benchmark demonstrate that Pov9D achieves state-of-the-art performance, improving Abs IoU@50 by 7.2% and Rel 10° 10 cm by 27.2% over OV9D.

## 1. Introduction

Open-vocabulary perception has become a key objective in 3D scene understanding, enabling models to recognize and reason about objects beyond a predefined category set. This capability is essential for building scalable and adaptive systems that can operate in dynamic, open-world environments, where the diversity of object types far exceeds the scope of closed-set training data. Recent advances have demonstrated promising results in open-vocabulary 3D object detection [1,2,3] and segmentation [4,5,6], making it possible to identify a wider range of categories specified by language inputs. However, most of these efforts are limited to recognition-level tasks, leaving further challenges in spatially aware understanding largely unexplored. One critical challenge in spatially aware understanding is object pose estimation, which involves accurately determining an object’s position, orientation, and size in 3D space. This capability is essential for a wide range of applications, including robotics [7,8,9] and augmented reality [10,11].

Existing object pose estimation approaches can be broadly categorized into three groups: (1) instance-level methods [12,13,14,15], which require CAD models for each object and are confined to closed-set scenarios; (2) category-level methods [16,17], which aim to generalize to unseen instances within a predefined set of categories, but still operate under fixed category assumptions; and (3) unseen object pose estimation methods [18,19,20,21], which attempt to handle novel categories at test time, often relying on strong priors such as CAD models, synthetic pretraining, or a small number of labeled exemplars. However, these methods are not well-suited to open-vocabulary scenarios, limiting their applicability in real-world settings. To address the limited generalizability of prior methods, a recent work [22] attempts to generalize pose estimation to unseen categories by predicting normalized object coordinates (NOCS) from RGB images and category-level text. However, this method depends heavily on appearance features and lacks the ability to reason over 3D geometry, resulting in limited performance when generalizing to novel shapes or categories.

**Figure 1:**
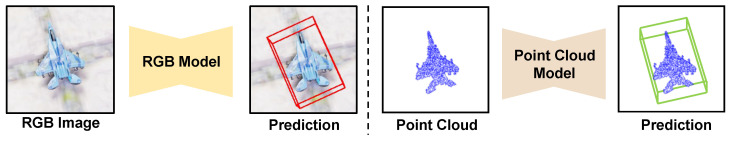
(**Left**) RGB-based baseline. (**Right**) Our point cloud-based method.

Unlike the existing method [22] that relies solely on RGB inputs, we introduce Pov9D, the first *point cloud-based* framework for open-vocabulary object pose estimation, as illustrated in Figure 1. In contrast to RGB-based approaches that primarily depend on appearance cues such as texture, color, and shading, Pov9D leverages explicit geometric signals from 3D point clouds, including surface curvature, object topology, and spatial structure. Such structural information, which is often ambiguous or lost in 2D projections, provides stronger constraints for recovering orientation and spatial alignment. This design choice makes Pov9D particularly effective for textureless or geometrically complex objects, as well as in cases where RGB lacks sufficient discriminative features, situations that typically lead to severe pose ambiguities for appearance-only methods. Moreover, point clouds are inherently invariant to appearance variations. When combined with a frozen large-scale 3D backbone, this property further enables open-world generalization without overfitting to category-specific textures, yielding accurate 9D pose estimation for both seen and unseen categories.

To further enhance point cloud-based open-vocabulary pose estimation performance, we introduce a language-conditioned shape prior that bridges geometry and semantics for generalizability. Instead of relying on fixed, category-specific priors, Pov9D synthesizes a normalized shape from a category-level text description (category semantics) and global 3D features (observed point cloud geometry). Specifically, a text- and geometry-conditioned generator outputs a normalized point cloud prior and a compact prior representation that conditions the decoder. This dynamic prior provides a stable geometric reference to guide NOCS and size prediction, mitigating pose ambiguities for textureless or geometrically complex objects and enabling category-agnostic 9D pose estimation.

In summary, the main contributions of this work are:

- Unlike existing RGB-based methods [22], we propose the first point cloud-based method for open-vocabulary 9D pose estimation. Point clouds capture fine-grained geometric structures such as surface curvature and spatial layout, which are often ambiguous in 2D images, and provide inherent robustness to variations in lighting, surface texture, and background clutter.
- We propose a language-conditioned shape prior generation module, which synthesizes category-level shape representations to guide geometry-aware alignment for unseen objects.
- Our method achieves state-of-the-art performance on the OO3D-9D benchmark, with substantial improvements across metrics. For example, it achieves 95.0% Abs IoU@50 compared to 87.8% by OV9D, and 71.6% accuracy under the Rel 10°10 cm setting versus 44.4% by OV9D.

## 2. Related Work

Object pose estimation focuses on recovering an object’s 3D position and orientation (6D) and, optionally, its size (to form 9D), serving as a critical technology for applications such as robotic grasping [7,8,9] and augmented reality [10,11]. However, most existing pose estimation methods are developed under a closed-set setting, where object categories are predefined or strong priors such as CAD models or category-specific training are required. These requirements limit their applicability in dynamic and multifaceted real-world environments.

*Instance-Level Pose Estimation.* Instance-level methods [12,15,23] constitute the classical paradigm of object pose estimation. These approaches assume that an accurate CAD model is available for each object during both training and testing, and the task is to establish precise correspondences between the observed input (RGB or RGB-D) and the given model. By exploiting rich geometric and texture information, they can achieve very high accuracy under controlled conditions. Their effectiveness under controlled conditions is demonstrated by methods such as SSD-6D [12], which integrates 2D detection with pose refinement; PoseCNN [23], which directly regresses poses from images; and SurfEmb [15], which leverages continuous surface embeddings for robust correspondence estimation. Despite these strengths, the heavy reliance on object-specific CAD models prevents them from generalizing to novel instances or categories, which severely limits their applicability in open and dynamic real-world environments.

*Category-Level Pose Estimation.* To relax the dependency on object-specific CAD models, category-level pose estimation methods aim to generalize across unseen instances within a predefined set of categories. A pioneering work is Normalized Object Coordinate Space (NOCS) [16], which establishes a shared normalized coordinate system for each category, allowing consistent alignment between observed objects and normalized shapes. Shape prior deformation [17] further introduces category-level shape priors and learns to deform them to better fit individual instances, improving accuracy under intra-class variation. Building on this idea, SGPA [24] leverages structure-guided priors for alignment, HS-Pose [25] incorporates hybrid-scope feature extraction to enhance geometric reasoning, and IST-Net [26] proposes implicit space transformations to achieve prior-free estimation. These methods constitute an important advancement, as they allow models to generalize from known exemplars to novel intra-category instances. However, their dependence on a predefined category set fundamentally limits scalability and renders them incapable of handling categories that were not observed during training.

*Unseen Object Pose Estimation.* To move beyond fixed categories, unseen object pose estimation aims to handle novel classes at test time. Early works such as multi-path learning [20] improved robustness across domains. Template-based [18] and retrieval-based methods [21] explored exemplar matching and local geometric similarities, while large-scale systems like MegaPose [27] leveraged synthetic pretraining and extensive 3D assets. More recent advances include diffusion and feature aggregation strategies [19]. FoundationPose [28] further unified pose estimation and tracking for novel objects. Despite these efforts, most approaches still rely on strong priors such as CAD models, synthetic data, or labeled references, limiting their scalability in real-world scenarios.

*Open-Vocabulary Object Pose Estimation.* Recent work [22] introduces OV9D, the first attempt at open-vocabulary 9D pose estimation. By leveraging category-level text embeddings and predicting normalized object coordinates from RGB images, OV9D enables pose estimation for unseen categories. However, since RGB inputs primarily capture appearance cues such as texture and color, OV9D suffers from severe pose ambiguity when dealing with textureless surfaces or instances lacking distinctive visual features. Recent multimodal approaches fall into two related lines: (i) 3D–vision–language alignment that learns a shared embedding space across 3D data and image/text modalities (e.g., OpenShape [29], ULIP [30]); and (ii) 3D-language models that couple a point-cloud encoder with an LLM for instruction following and semantic grounding (e.g., PointLLM [31]). These methods focus on recognition, retrieval, or language-driven reasoning rather than estimating full 9D object poses (position, orientation, and scale). Point cloud learning has also been explored in physical modeling [32,33]. In contrast, we introduce Pov9D, the first point cloud-based framework for open-vocabulary 9D pose estimation. Unlike RGB-based approaches, Pov9D directly leverages 3D geometric structures, which provide strong cues for disambiguating textureless surfaces or instances lacking distinctive visual features. Moreover, we introduce a text-conditioned shape prior generator, inspired by generative point cloud models such as Point-E [34], to dynamically synthesize shape priors for unseen categories. This design enables Pov9D to achieve robust pose estimation without relying on CAD models or category-specific training, thereby fully realizing the potential of open-vocabulary generalization.

## 3. Method

Point cloud-based representations provide explicit 3D surface geometry, which is crucial for accurate object pose estimation in open-vocabulary settings. Unlike prior RGB-based approaches [22], which rely on appearance cues such as texture and color, point clouds directly capture metric surfaces and local differential structures, including curvature, edges, and planar patches, providing strong geometric constraints that reduce perspective ambiguity and help disambiguate textureless or geometrically complex objects. Consequently, this representation enables robust reasoning about object translation, orientation, and scale.

Motivated by these advantages, we propose Pov9D, a point cloud-based framework that estimates object size $s\in R^{3}$, translation $t\in R^{3}$, and orientation R∈SO(3) directly from an observed point cloud P and a category-level text description *T*. The input point cloud $P={\left\{p_{i}\in R^{3}\right\}}_{i=1}^{N}$ consists of *N* points, and the estimation process can be expressed as:

(1) (R,t,s)=Pov9D(P,T).

**Figure 2:**
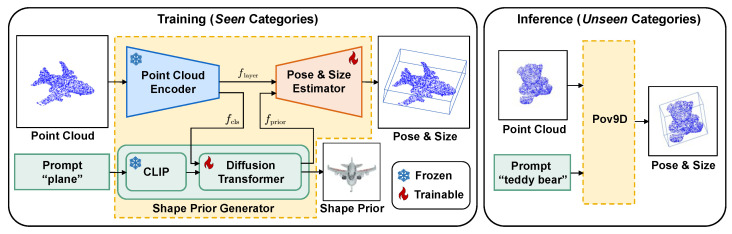
Overview of the Pov9D framework for open-vocabulary 9D object pose estimation. (**Left**) **Training on** ***seen*** **categories (e.g., plane).** Given a point cloud and its category-level text description, the frozen point cloud encoder (Section 3.1) extracts features $f_{\mathrm{layer}}$ and $f_{\mathrm{cls}}$. The normalized shape prior generator (Section 3.2) uses both text and global 3D features to produce a normalized shape prior $f_{\mathrm{prior}}$. The pose and size estimator (Section 3.3) decodes $f_{\mathrm{layer}}$ and $f_{\mathrm{prior}}$ to predict the pose and size. (**Right**) **Inference on** ***unseen*** **categories (e.g., teddy bear).** The Pov9D generalizes to novel object categories without retraining, leveraging the learned alignment between text, shape prior, and geometry.

The proposed Pov9D framework is illustrated in Figure 2, which consists of three main modules: the point cloud encoder (Section 3.1), which extracts geometric and semantic features from the input point cloud; the normalized shape prior generator (Section 3.2), which synthesizes a category-level normalized shape conditioned on text and geometry; and the pose and size estimator (Section 3.3), which predicts *R*, *t*, and *s*.

### 3.1. Point Cloud Encoder

In contrast to RGB-based method [22], our approach directly operates on 3D point clouds, leveraging their geometric richness for more accurate prediction. To further enhance generalizability, we adopt a pretrained Uni3D [35] encoder with a ViT-G [36] backbone to extract geometric and semantic features from the input (Figure 2). Unlike prior works on closed-set category-level object pose estimation [25,26], which typically train point cloud encoders from scratch, we leverage a large-scale pretrained 3D backbone and keep it frozen during training, reducing overfitting and providing robust, transferable representations for novel and unseen categories. This encoder not only captures local geometry but also encodes global semantics. The encoder produces a global CLS token $f_{\mathrm{cls}}$ that encodes high-level semantic information, which we use to condition the generation of the normalized shape prior (Section 3.2). Additionally, we utilize layer-wise features $f_{\mathrm{layer}}$ extracted from the 10th, 20th, 30th, and final (40th) encoder transformer layers, which preserve dense spatial and geometric cues to facilitate the prediction of the normalized object coordinate space (NOCS) map and object size (Section 3.3):

(2) $$\left\{f_{\mathrm{cls}},f_{\mathrm{layer}}\right\}=\mathrm{Enc}\left(P\right).$$

### 3.2. Normalized Shape Prior Generator

In open-vocabulary object pose estimation, the absence of 3D models for novel objects makes it difficult to infer accurate pose and size. To overcome this, we introduce a normalized shape prior generator that predicts a shape prior from the input point cloud and its associated text description, as shown in Figure 2. This predicted prior offers strong geometric cues for downstream pose and size estimation. Specifically, this module is based on a text-conditional point cloud generation model [34]. We modify two key components: (1) In addition to the original text-based conditioning $f_{\mathrm{text}}$ (obtained by encoding the category-level description *T* using a pretrained CLIP text encoder [37]), we incorporate the CLS token $f_{\mathrm{cls}}$ output from the encoder as an additional conditioning input, providing richer semantic and geometric context. (2) Unlike the original multi-step denoising process, we adopt a single-step generation strategy that directly maps noise to a point cloud:

(3) $$\left[\hat{S},f_{\mathrm{prior}}\right]=G\left(z|f_{\mathrm{text}},f_{\mathrm{cls}}\right),$$

where z∼N(0,I) is a random noise vector, and *G* denotes the conditional generator. The generator outputs a normalized shape prior point cloud $\hat{S}\in R^{N\times 3}$. These enhancements significantly improve generation efficiency while maintaining the geometric and semantic fidelity of the generated normalized shape prior.

To provide a consistent reference frame and remove scale ambiguity, we adopt a category-agnostic normalized coordinate system in which shapes are centered at the origin and isotropically scaled to unit extent. Given a ground-truth shape $S=\{x_{j}\}$, we compute the centroid and scale:

(4) $$\mu_{S}=\frac{1}{\left|S\right|}\sum_{x\in S}x,\;r_{S}=\max_{x\in S}{∥x-\mu_{S}∥}_{2},\;\bar{S}=\left\{\frac{x-\mu_{S}}{r_{S}}\;|\;x\in S\right\},$$

where $\mu_{S}\in R^{3}$ is the centroid, $r_{S}\in R_{+}$ is the isotropic scale (maximum radius), and $\bar{S}$ is the normalized shape. Thus, both $\hat{S}$ and $\bar{S}$ lie inside the unit ball and are centered at the origin. For symmetric categories, the frame is defined up to the symmetry group (e.g., rotations around the symmetry axis).

We train the shape prior generator using a combined loss:

(5) $$L_{\mathrm{shape}}=L_{\mathrm{CD}}\left(\hat{S},\bar{S}\right)+\lambda_{\mathrm{rep}}\cdot L_{\mathrm{rep}}\left(\hat{S}\right),$$

where $L_{\mathrm{CD}}$ is the Chamfer Distance [38] computed in the normalized space between predicted $\hat{S}$ and normalized ground truth $\bar{S}$, and $L_{\mathrm{rep}}$ promotes uniformity by penalizing closely packed points:

(6) $$L_{\mathrm{rep}}\left(\hat{S}\right)=\frac{1}{Nk}\sum_{i=1}^{N}\sum_{j\in N_{k}\left(i\right)}\exp\left(-\frac{∥{\hat{p}}_{i}-{\hat{p}}_{j}{∥}^{2}}{h^{2}}\right),$$

where *N* is the number of points, *h* is a bandwidth parameter, and $N_{k}\left(i\right)$ is the set of *k* nearest neighbors of point ${\hat{p}}_{i}$. We use the final-layer feature of the generator as the shape prior representation $f_{\mathrm{prior}}$.

### 3.3. Pose and Size Estimator

This module is responsible for estimating the 9D object pose, including rotation *R*, translation *t*, and size *s*. To achieve this, it predicts a normalized object coordinate space (NOCS) map ${\hat{m}}_{\mathrm{nocs}}$, which is used to recover *R* and *t* via the Umeyama algorithm [39] within a RANSAC framework, while the object size *s* is regressed in parallel. Specifically, the decoder takes $f_{\mathrm{layer}}$ and $f_{\mathrm{prior}}$ as inputs. It first upsamples $f_{\mathrm{layer}}$ using feature propagation [40], then concatenates it with $f_{\mathrm{prior}}$ to incorporate shape prior information. The decoder consists of three shared MLP layers with LeakyReLU [41] activation, followed by two parallel branches: the NOCS branch is an MLP that outputs the per-point NOCS map, while the size branch applies global average pooling followed by an MLP to regress the object size:

(7) $$\left({\hat{m}}_{\mathrm{nocs}},s\right)=\mathrm{Decoder}\left(f_{\mathrm{layer}},f_{\mathrm{prior}}\right).$$

Following [22], we adopt a Smooth L1 loss to supervise the NOCS prediction:

(8) $$L_{\mathrm{nocs}}=\frac{1}{N}\sum {i=1}^{N}\sum_{c\in x,y,z}{\mathrm{smooth}}_{\beta }\left({\hat{m}}_{\mathrm{nocs}}^{\left(i,c\right)}-m_{\mathrm{nocs}}^{\left(i,c\right)}\right),$$

where ${\hat{m}}_{\mathrm{nocs}}\in R^{N\times 3}$ is the predicted per-point NOCS map, $m_{\mathrm{nocs}}\in R^{N\times 3}$ is the ground truth, *N* is the number of points, and β>0 is the transition parameter (default β=1):

(9) $${\mathrm{smooth}}_{\beta }\left(u\right)=\left\{\begin{array}{cc} \frac{1}{2\beta }u^{2}, & \mathrm{if}\;|u|<\beta ,\\ \left|u\right|-\frac{\beta }{2}, & \mathrm{otherwise}. \end{array}\right.$$

In addition, the object size prediction is supervised using an L2 loss:

(10) $$L_{\mathrm{size}}=\frac{1}{N}\sum {i=1}^{N}{\left(s_{i}-{\hat{s}}_{i}\right)}^{2},$$

where $s_{i}$ is the ground-truth size, and ${\hat{s}}_{i}$ is the predicted size.

The total loss is given by:

(11) $$L_{\mathrm{total}}=L_{\mathrm{nocs}}+\lambda_{\mathrm{shape}}L_{\mathrm{shape}}+\lambda_{\mathrm{size}}L_{\mathrm{size}},$$

where $\lambda_{\mathrm{shape}}$ and $\lambda_{\mathrm{size}}$ are weighting factors to balance the contributions of losses, respectively.

## 4. Experiments

### 4.1. Experiment Settings

*Training Details.* We train the model for 25 epochs using the AdamW [42] optimizer with a learning rate of $1\times 10^{-4}$ and a cosine annealing scheduler. Training is performed with a batch size of 32 on two NVIDIA A6000 GPUs (Santa Clara, CA, USA). Both the input point cloud and the ground-truth shape prior are uniformly downsampled to 2048 points for efficiency. For fair comparison, we follow the protocol in [22] and use the ground-truth instance masks provided in the dataset, which can also be reasonably obtained using recent open-vocabulary 3D segmentation models [43,44]. To balance the training objectives, we set the loss weights to $\lambda_{\mathrm{shape}}=1$, $\lambda_{\mathrm{size}}=5$, and $\lambda_{\mathrm{rep}}=0.01$.

*Dataset.* Following [22], we evaluate our model on the OO3D-9D benchmark, which contains 5371 object instances across 216 diverse categories, covering a wide range of non-symmetric, discrete symmetric, and continuous symmetric objects. The dataset provides large-scale photorealistic RGB-D scenes that are particularly suitable for assessing open-vocabulary object pose estimation, as it enables testing on categories not seen during training. In our experiments, we follow the single-object setting and manually select 10 unseen categories, yielding 230 object instances that include both everyday items and geometrically challenging shapes. This test split serves as a rigorous benchmark for evaluating the generalization ability of our method to novel object categories.

### 4.2. Evaluation Metrics

We adopt three complementary metrics: (1) 3D IoU [45]. This metric measures the overlap between predicted and ground-truth 3D bounding boxes via Intersection-over-Union. We report axis-aligned IoU@50 and regard a prediction as correct when IoU≥0.5. (2) Absolute rotation-and-translation precision [46]. We denote the threshold setting as a° b cm. This metric counts the proportion of samples whose rotation error is within *a* degrees and whose translation error is within *b* centimeters. For objects with discrete symmetries, evaluation is conducted against all symmetry-equivalent ground-truth poses and the minimum error is used; for continuous symmetries, rotation is evaluated modulo the symmetric axis by checking principal-axis agreement. (3) Relative rotation-and-translation precision [22]. For unseen categories, the model may adopt an object reference frame different from the dataset’s normalized one, making absolute errors less indicative. This metric evaluates the internal consistency of predictions within a category by comparing poses relatively: for each category, treat each instance as an anchor, form relative poses from that anchor to all other instances in both predictions and ground truth, and count the fraction whose relative rotation and translation fall within the same a° and b cm thresholds; the category score is the best consistency over all anchors. Symmetries are handled as above. We report Abs IoU@50 and Abs 5° 5 cm/10° 5 cm/10° 10 cm, together with their relative counterparts (Rel 5° 5 cm, Rel 10° 5 cm, Rel 10° 10 cm).

### 4.3. Results on OO3D-9D

We evaluate our method on the OO3D-9D dataset for both absolute and relative pose estimation tasks. As shown in Table 1 and Table 2, Pov9D consistently outperforms all baselines, including PCA [47], IST [26], and OV9D [22], across a range of metrics. For absolute pose estimation, our method achieves an average IoU@50 of 95.0%, surpassing OV9D, which reaches 87.8%, by a significant margin. In the more fine-grained 5° 5 cm setting, we improve accuracy from 15.8% to 24.2%. For relative pose estimation, Pov9D similarly shows strong gains, achieving 71.6% under the 10° 10 cm criterion compared to 44.4% by OV9D. These improvements are most pronounced on textureless or geometrically complex objects such as bumbags, facial cream, and dumplings, where existing methods often struggle due to inherent pose ambiguities. These results highlight the key strengths of our approach. Pov9D explicitly leverages geometric information from point clouds and integrates learned shape priors to enhance pose prediction. By directly operating on 3D data, our model captures fine-grained surface structure that RGB-based methods typically miss, enabling more accurate alignment between observed instances and normalized shapes. The normalized shape prior generator further contributes by providing strong structural cues, allowing the model to generalize to novel categories and resolve ambiguities in the absence of texture or distinctive appearance. Together, these components lead to robust and accurate open-vocabulary object pose estimation across a wide variety of object types.

**Figure 3:**
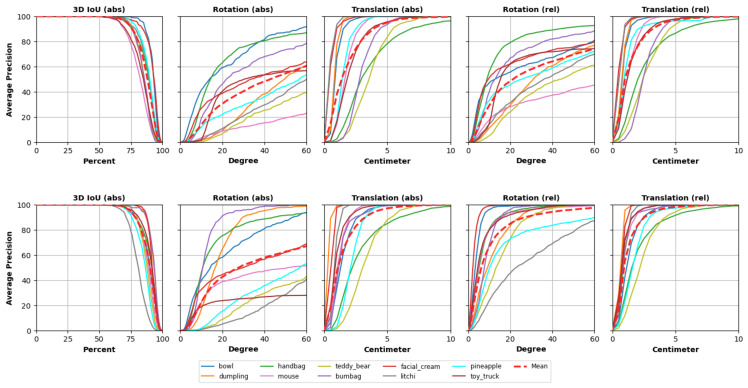
Comparison of absolute and relative precision on the OO3D-9D benchmark. Top: OV9D [22]; bottom: Pov9D (ours). Average precision (AP) is reported for varying thresholds of 3D IoU, rotation, and translation. Pov9D consistently outperforms OV9D across all thresholds.

Figure 3 visualizes absolute and relative precision as average precision (AP) curves with respect to 3D IoU, rotation, and translation thresholds (top: OV9D; bottom: Ours). Our method yields consistently higher AP across thresholds.

### 4.4. Ablation Study

To assess key components, we perform ablations on the OO3D-9D benchmark, focusing on (1) textual input and shape prior design, and (2) encoder pretraining.

*Effect of Text Input and Prior Design* Table 3 shows an ablation on text input and prior design. Removing text reduces Abs 5° 5 cm to 22.3 from 24.2, highlighting the importance of category-level semantics. We focus on Abs 5° 5 cm as the most stringent metric. CLIP embeddings improve it to 23.6, unnormalized priors to 23.4, both below the full model. Our full model, combining text-guided and normalized priors, achieves 24.2.

*Effect of Encoder Pretraining* Table 4 shows that training the encoder from scratch yields the weakest Abs 5° 5 cm of 15.6. We focus on Abs 5° 5 cm as the most stringent metric. Pretraining with Uni3D improves it to 22.8, and freezing the pretrained encoder reaches 24.2, showing that pretrained 3D features generalize well to open-vocabulary pose tasks.

### 4.5. Visualization

**Figure 4:**
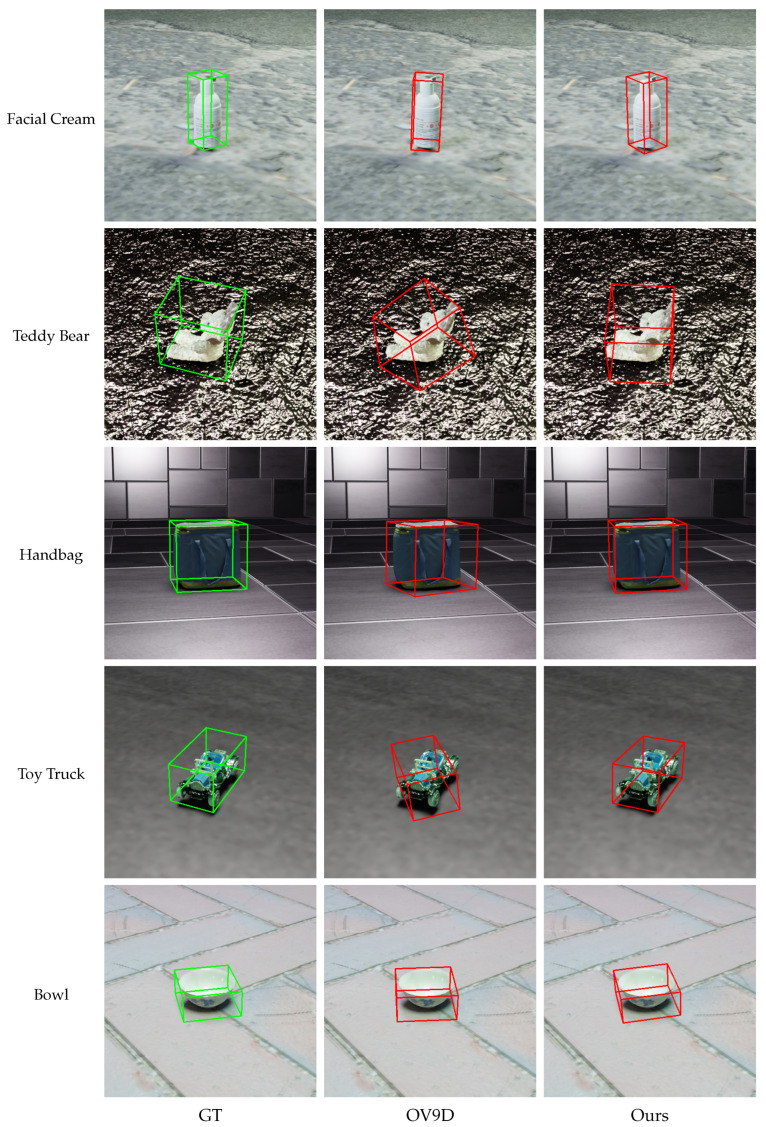
Qualitative comparison of pose estimation results on representative object categories. Each row shows a different object instance, including intricate or irregular geometric structures (e.g., toy truck, handbag) and textureless items (e.g., facial cream). For each case, we visualize the ground truth pose (GT), the OV9D baseline prediction, and our Pov9D prediction. Please add an explanation for red and green box in the figure. Red boxes indicate predicted poses, while green boxes indicate GT.

Figure 4 presents qualitative comparisons between our Pov9D method and the OV9D baseline across a variety of object categories, including textureless or geometrically complex objects. For each instance, we visualize the ground truth pose (GT), the OV9D prediction (OV9D), and our method’s prediction (Ours). As shown, Pov9D consistently produces more accurate and robust pose estimations, with consistently superior alignment on challenging objects, including those with intricate or irregular geometric structures (e.g., toy truck, handbag) and textureless items (e.g., facial cream). Compared to OV9D, our approach yields improved rotation, translation, and scale estimation, benefiting from explicit geometric reasoning and the integration of language-conditioned shape priors. These visual results highlight Pov9D’s superior generalization and reliability in open-vocabulary 9D pose estimation, especially for objects with complex shapes or ambiguous appearance.

**Figure 5:**
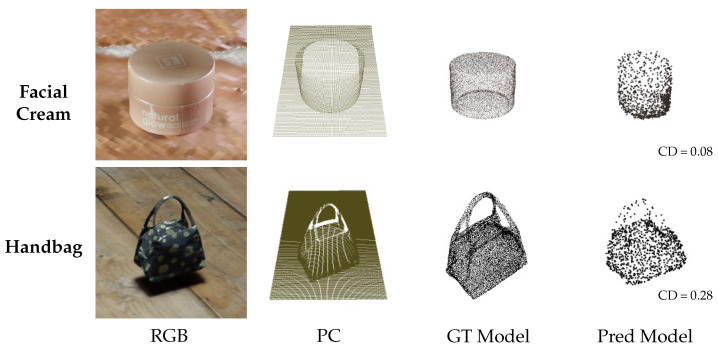
Qualitative validation of the normalized shape prior generator. The figure shows two object categories (Facial Cream, Handbag) and four data modalities: RGB image (for visualization only, not used as model input), point cloud, ground truth model, and predicted model. The numbers in the predicted models indicate the Chamfer Distance (CD) errors.

To better illustrate the effectiveness of the normalized shape prior generator, Figure 5 visualizes its outputs for two object categories (facial cream, handbag) in four modalities, including the RGB image, the point cloud, the ground truth model, and the predicted model. The RGB image is shown only for visualization. This figure highlights how the generator produces normalized shape priors that closely match the ground truth geometry, validating its contribution to accurate pose estimation. The corresponding Chamfer Distance (CD) errors are also reported, further confirming the geometric consistency between the generated and ground truth shapes.

### 4.6. Ablation on Loss Weight

To assess the effect of loss balancing, we conduct experiments over $\lambda_{\mathrm{shape}}\in \left\{0.5,1.0,2.0\right\}$ and $\lambda_{\mathrm{size}}\in \left\{2,5,8\right\}$, centered at the default (1,5). As shown in Table 5, the performance remains largely consistent across all configurations, indicating limited sensitivity to loss weighting.

### 4.7. Latency

The average inference time is 90 ms per sample (about 11FPS) on an A6000 GPU, which provides near real-time performance sufficient for interactive robotic perception and AR prototyping.

## 5. Discussion

In this section, we analyze the limitations of our approach, with the primary challenge arising from low-resolution and partial point clouds. In practice, commodity depth sensors often produce point clouds that are sparse, noisy, and truncated, especially for thin parts, glossy surfaces, or distant objects that are difficult to capture reliably. Moreover, small extrinsic or intrinsic calibration errors can further enlarge geometry gaps, erasing edges and surface cues that are critical for establishing accurate correspondences. These imperfections make orientation and scale estimation unstable under strict thresholds, with errors particularly concentrated on thin or partially visible objects. To quantify these effects, we conduct controlled robustness experiments under Gaussian noise perturbation and partial occlusion, as summarized in Table 6 and Table 7. The results show that moderate noise or missing regions cause only limited degradation, while severe corruption (e.g., 0.2 noise level or 20% occlusion) leads to noticeable drops in both absolute and relative pose metrics. This confirms that our method maintains reasonable robustness under realistic sensor imperfections but remains sensitive to strong geometric incompleteness. As a future research direction, multimodal fusion that integrates high-resolution RGB and depth with point clouds could be explored to enrich geometric representation and improve the robustness of pose estimation.

While our method benefits from text-conditioned priors, it may fail when the language input is ambiguous or under-specified. For example, phrases referring to multiple object variants (“mug” vs. “cup”) can lead to mismatched shape priors and degraded pose accuracy. Future work could address this issue by incorporating more context-aware text embeddings or grounding mechanisms to disambiguate category semantics.

## 6. Conclusions

In this paper, we propose Pov9D, a point cloud-based framework for open-vocabulary 9D object pose estimation. Unlike RGB-based methods that rely on 2D appearance cues, Pov9D directly exploits 3D geometry to estimate rotation, translation, and size from a single scan and a category-level text prompt. To enhance generalization in open-vocabulary scenarios, we introduce a language-conditioned normalized shape prior that integrates category information and observed point cloud features, serving as a normalized geometric reference to guide NOCS and size estimation. Extensive experiments on OO3D-9D validate these advantages: Pov9D achieves state-of-the-art results across absolute and relative pose metrics, with large gains on textureless or geometrically complex objects where RGB methods struggle. Ablations further confirm the benefit of the language-guided prior and the frozen large-scale 3D backbone in enabling open-world generalization. Future research will investigate multimodal fusion with RGB-D, the development of more powerful normalized shape prior generators, and the incorporation of finer-grained textual conditioning, with the goal of enhancing robustness under sparse or partial observations and strengthening the generalization capacity in open-vocabulary settings.

## Figures and Tables

**Table 1:** Absolute pose estimation results on OO3D-9D. †: continuous symmetric, ‡: discrete symmetric, *: non-symmetric. The best result in each column is highlighted in bold.

	Abs IoU@50				Abs 5° 5 cm				Abs 10° 5 cm				Abs 10° 10 cm			
	**PCA**	**IST**	**OV9D**	**Ours**	**PCA**	**IST**	**OV9D**	**Ours**	**PCA**	**IST**	**OV9D**	**Ours**	**PCA**	**IST**	**OV9D**	**Ours**
bowl †	19.2	53.7	**97.3**	94.0	1.2	14.7	41.7	**41.7**	1.3	32.5	**62.3**	59.0	1.3	32.5	**62.3**	59.1
bumbag ‡	29.6	40.4	91.7	**97.7**	2.4	0.0	10.9	**28.0**	5.2	0.2	41.5	**73.3**	5.8	0.2	41.5	**73.3**
dumpling ‡	46.7	64.5	81.2	**98.2**	3.7	0.0	1.8	**10.5**	6.6	0.4	11.0	**37.5**	6.6	0.4	11.0	**37.5**
facial cream ‡	3.6	37.1	91.3	**99.8**	0.4	7.7	34.0	**44.6**	1.7	17.1	52.7	**65.0**	1.7	17.1	52.7	**65.0**
handbag ‡	35.5	52.3	91.8	**97.3**	1.6	0.0	22.0	**37.8**	3.0	0.2	54.7	**71.5**	4.1	0.2	56.0	**71.7**
litchi †	32.4	89.9	94.6	**94.8**	1.7	**15.3**	7.0	8.6	2.8	26.4	18.6	**22.6**	2.8	**26.4**	18.6	22.6
mouse *	10.8	28.8	72.9	**93.1**	0.3	0.0	6.3	**20.7**	0.8	0.1	17.7	**46.0**	0.8	0.1	17.7	**46.0**
pineapple †	29.7	41.0	**89.4**	86.6	1.4	6.9	**30.8**	12.7	2.8	14.2	**49.5**	34.2	3.2	15.0	**49.5**	34.2
teddy bear *	64.0	61.8	91.6	**92.0**	0.2	0.0	1.0	**5.0**	0.4	0.6	5.0	**20.8**	0.6	0.6	5.2	**20.8**
toy truck *	58.7	57.5	76.5	**96.0**	5.9	0.0	2.8	**25.5**	9.6	0.0	11.3	**39.5**	9.6	0.0	11.3	**39.5**
**Average**	33.2	52.7	87.8	**95.0**	1.9	4.5	15.8	**24.2**	3.4	9.2	32.4	**47.8**	3.7	9.3	32.6	**47.8**

**Table 2:** Relative pose estimation results on OO3D-9D. †: continuous symmetric, ‡: discrete symmetric, *: non-symmetric. The best result in each column is highlighted in bold.

	Rel 5° 5 cm				Rel 10° 5 cm				Rel 10° 10 cm			
	**PCA**	**IST**	**OV9D**	**Ours**	**PCA**	**IST**	**OV9D**	**Ours**	**PCA**	**IST**	**OV9D**	**Ours**
bowl †	2.3	1.0	66.3	**67.6**	4.7	2.7	90.5	**94.0**	9.3	2.7	90.5	**94.5**
bumbag ‡	2.4	0.2	14.2	**27.7**	4.0	0.2	44.2	**74.6**	11.8	0.2	44.2	**74.6**
dumpling ‡	6.0	0.1	1.9	**11.0**	11.3	0.1	10.4	**38.7**	11.3	0.1	10.4	**38.7**
facial cream ‡	2.1	0.3	25.3	**85.6**	4.9	2.3	56.4	**99.2**	4.9	2.3	58.4	**99.2**
handbag ‡	4.1	0.0	25.3	**38.1**	6.8	0.1	56.4	**71.2**	10.0	0.1	56.4	**71.6**
litchi †	2.4	0.4	**18.4**	15.8	3.7	2.5	**40.5**	40.1	3.7	2.5	**40.5**	40.1
mouse *	2.7	0.1	3.9	**19.6**	7.3	0.1	15.0	**45.5**	7.3	0.1	15.0	**45.5**
pineapple †	1.9	0.2	**47.3**	28.0	3.4	0.8	**69.0**	53.1	3.4	0.8	**72.3**	53.7
teddy bear *	2.0	0.2	1.0	**3.2**	3.0	0.2	2.0	**16.2**	4.8	0.2	2.0	**16.2**
toy truck *	2.2	0.1	4.2	**10.4**	5.0	0.2	12.9	**36.4**	5.0	0.2	12.9	**36.4**
**Average**	2.8	0.3	26.8	**45.3**	5.4	0.9	43.8	**71.0**	7.2	0.9	44.4	**71.6**

**Table 3:** Ablation study on textual input and prior design. The best result in each column is highlighted in bold.

Text Input	Abs IoU@50	Abs 5° 5 cm	Abs 10° 10 cm
w/o Text	93.2	22.3	45.6
CLIP Embedding	93.5	23.6	46.1
Unnormalized Prior	94.1	23.4	47.0
Ours (Full)	**95.0**	**24.2**	**47.8**

**Table 4:** Ablation study on encoder initialization and training strategy. The best result in each column is highlighted in bold.

Encoder Strategy	Abs IoU@50	Abs 5° 5 cm	Abs 10° 10 cm
Scratch (Finetuned)	85.1	15.6	31.4
Pretrained (Finetuned)	93.2	22.8	46.2
Pretrained (Frozen)	**95.0**	**24.2**	**47.8**

**Table 5:** Sensitivity analysis on loss weights $\lambda_{\mathrm{shape}}$ and $\lambda_{\mathrm{size}}$ The best result in each column is highlighted in bold.

$\lambda_{\mathrm{shape}}$	$\lambda_{\mathrm{size}}$	IoU@50	5° 5 cm	10° 10 cm
0.5	2	94.1	23.5	46.3
0.5	5	94.6	23.9	47.0
0.5	8	94.2	23.6	46.5
1.0	2	94.3	23.8	47.1
1.0	5	**95.0**	**24.2**	**47.8**
1.0	8	94.5	23.7	47.0
2.0	2	94.0	23.3	46.1
2.0	5	94.7	24.0	47.4
2.0	8	94.3	23.5	46.8

**Table 6:** Robustness analysis under Gaussian noise perturbation. The best result in each column is highlighted in bold.

Noisy Level	Abs IoU@50	Abs 5° 5 cm	Abs 10° 10 cm
0.	**95.0**	**24.2**	**47.8**
0.1	94.2	22.9	46.9
0.2	91.1	19.6	45.1

**Table 7:** Robustness analysis under partial occlusion. The best result in each column is highlighted in bold.

Occlusion Rate	Abs IoU@50	Abs 5° 5 cm	Abs 10° 10 cm
0%	**95.0**	**24.2**	**47.8**
10%	93.6	23.1	47.0
20%	90.3	20.3	45.2

## Data Availability

The oringinal data presented in the study are openly avalible in [OmniObject3D dataset] at [https://opendatalab.com/OpenXD-OmniObject3D-New/download( accessed on 25 October 2025)] and [the OV9D dataset] at [https://github.com/caijunhao/ov9d( accessed on 25 October 2025)].
